# Equity in Out-of-Pocket Payments for Healthcare Services: Evidence from Malaysia

**DOI:** 10.3390/ijerph19084500

**Published:** 2022-04-08

**Authors:** Mohamed Fakhri Abu Baharin, Muhamad Hanafiah Juni, Rosliza Abdul Manaf

**Affiliations:** 1 Public Health Unit, Department of Primary Health Care, Faculty of Medicine and Health Sciences, Universiti Sains Islam Malaysia, Nilai 71800, Malaysia; 2 Department of Community Health, Faculty of Medicine and Health Sciences, Universiti Putra Malaysia, Serdang 43400, Malaysia; hanafiah_juni@upm.edu.my (M.H.J.); rosliza_abmanaf@upm.edu.my (R.A.M.)

**Keywords:** equity, progressivity, out-of-pocket payments, health equity, health expenditures, Malaysia, Kakwani index

## Abstract

Background: Out-of-pocket (OOP) payments are an inequitable mechanism for health financing as their high share of total health expenditures poses a risk of catastrophic healthcare expenditures. This study aimed to assess the distribution and progressivity of OOP payments made by Malaysian households for various group of healthcare services. Methods: This study utilized data from the Malaysian Household Expenditure Survey (HES) between 2014 and 2015, which involved 14,473 households. Distribution and progressivity of OOP payments were measured through their proportion of household consumption, a concentration curves plot and the Kakwani Progressivity Index (KPI). Results: The mean proportion of Malaysian OOP payments for healthcare of household consumption was 1.65%. The proportion increased across households’ consumption quintiles, from 1.03% made by the poorest 20% to 1.86% by the richest 20%. The OOP payments in Malaysia were progressive with a positive KPI of 0.0910. The OOP payments made for hospital-based services were the most progressive (KPI 0.1756), followed by medical products, appliances and equipment (KPI 0.1192), pharmaceuticals (0.0925) and outpatient-based services (KPI 0.0394) as the least progressive. Conclusions: Overall, the OOP payments for healthcare services in Malaysia were progressive and equitable as they were more concentrated among the richer households.

## 1. Introduction

Equity in healthcare funding and financial risk protection are important policy priorities of the healthcare system. The notion of equity or fairness in healthcare spending, according to the World Health Organization, is that healthcare should be provided according to the ability of households to pay, and they should not be burdened by healthcare expenditure to the degree that it has catastrophically decreased their welfare [1]. To this day, out-of-pocket (OOP) payments are one of the main sources of financing healthcare systems. Previous work has concentrated primarily on out-of-pocket (OOP) payments in the study of financial risk protection. Household OOP health payments are healthcare expenses charged directly to the budget of the household that are not reimbursed at the point of care, by public or private insurance or any third party [2]. High reliance on OOP health payments in the case of illness predisposes households to incurring large medical expenses, which pose a financing risk of ill health, thus leading households into financial catastrophe, particularly among the poorer households [3].

In 2019, almost 35% of Malaysia’s total health expenditure came from OOP payments, which fund 74% of Malaysia’s private healthcare services [4]. This figure was high compared to upper-middle income (32%) and Western Pacific Region (25.9%) countries’ standard [5]. Information on the share of OOP health spending of a country’s total financing alone does not provide a full picture of the degree to which such payments will jeopardize the welfare of households. The distribution of health payments from OOPs that fall disproportionately on poorer households can provide an indicator of the greater effect on financial equity on welfare. One of the tools to measure health financial equity is measuring the progressivity of health payments, which show the extent of inequality in paying for healthcare services between households of unequal living standards or ability to pay (ATP) [6,7]. Progressivity of OOP health payments varies around the world. Previous studies indicate the OOP payments were regressive in low-income countries such as in Nigeria [8] and lower-middle income countries such as Bangladesh [9] and India [10], indicating a high burden of OOP payments falls on the poor. Other developing middle-income countries, such as China and Iran, made fairly progressive OOP health payments [11,12,13].

There are limited studies done to study the progressivity of OOP health payments in Malaysia. One of the earlier studies to assess the progressivity of OOP health payments was carried out by using data from the Malaysian Household Expenditure Survey (HES) 1998/1999, which showed a slight progressivity of the payments [14,15]. Another similar Malaysian study compared the progressivity trend of OOP payments using three year rounds of Household Expenditure Surveys (HES), namely HES 1993/1994, HES 1998/1999 and HES 2004/2005 [16]. That study indicated the OOP payments for all three HES years were progressive, although the progressivity trends showed a declining pattern, which indicates the burden of OOP payments were increasingly felt by the poorer households. Given the substantial burden of how OOP payments can affect a household’s financial risk protection and the limited availability of local data, there is an urgent need to assess the equity of OOP payments for healthcare services made by Malaysian households. Thus, this study aimed to assess the equity of OOP payments through determining the distribution and progressivity of households’ OOP payments for different types of healthcare services, utilizing data from the Malaysian Household Expenditure Survey (HES) between 2014 and 2015 (HES 2014/2015).

## 2. Materials and Methods

### 2.1. Study Setting and Design

This was a retrospective, cross-sectional study which utilized secondary data from the Household Expenditure Survey (HES), conducted nationwide among Malaysian households between 2014 and 2015 by the Department of Statistics Malaysia (DOSM). The Malaysian HES was first conducted in 1973, and since then it has been carried out nationwide every five years, with HES 2014/2015 being the most recent data available at the time of study. This survey was conducted among the private households in all thirteen states and three federal territories of Malaysia, both in urban and rural areas. The HES survey collects data on twelve groups of household expenditure items, which are based on the international Classification of Individual Consumption According to Purpose (COICOP), developed by the United Nations. The twelve groups of household expenditure items range from purchases of food, transport and clothing to healthcare items or services, which was the main expenditure group of interest in this study.

### 2.2. Study Sample

This study involved a universal sampling method, in which all households sampled in the HES 2014/2015 were enrolled. A total of 14,437 households were sampled, for which a household was the sampling unit of analysis, instead of individual participants. A household in this study consisted of related and/or unrelated persons who usually live together and make common provisions for food and other essentials of living. Exclusion criteria included households of non-citizens of Malaysia, households who did not have expenditures on medical/health-related items or services and other groups of household expenditures that were not relevant to this study (non-medical/health-related household expenditures).

### 2.3. Study Variables

The two main variables used in this study were the OOP payments made by households to purchase healthcare services or items and the household’s ability to pay (ATP).

#### 2.3.1. Out-of-Pocket Payments for Healthcare

OOP payments refer to the expenses made by households to purchase or receive healthcare services or items that are paid with their own money or cash reserves that are not reimbursed by any third-party sources such as insurance companies or governmental aid. The OOP payments for healthcare services or items made by Malaysian households comprise all payments made for healthcare items listed in the household expenditure group 6: health expenditure. There are nine expenditure groups for different types of healthcare items or services in the HES 2014/2015, which are pharmaceutical products, therapeutic appliances and equipment, other medical products, medical services, dental services, paramedical services, government hospitals, government corporate hospitals and private hospitals.

For the purpose of analysis in this study, the OOP payments were grouped into four groups of interest, in terms of the broad functional use of the healthcare services or items. The groups were as follows:

(i) Pharmaceuticals: all payments made for prescriptive and non-prescriptive medicine, medicine for health upkeep, such as vitamins, health supplements, purchase of traditional and complementary medicine, etc.
(ii) Medical products, appliances and equipment: all payments made for health-related products or medical appliances, such as contraceptives, pregnancy test kits, vision aids, dentures, orthopedic braces, prostheses, face masks, etc.
(iii) Outpatient-based services: all payments incurred from outpatient treatment in government and private clinics, dental services and paramedical services, such as those for laboratory tests, as well as payments made for traditional or alternative medicine such as homeopathy, acupuncture, etc.
(iv) Hospital/inpatient-based services: all payments incurred from inpatient treatment in government or private hospitals, such as those from registration fees, ward charges, treatment fees including specialist/consultant fees, etc.

#### 2.3.2. Household’s Ability to Pay (ATP)

Household’s ability to pay (ATP) indicates the living standards or welfare of a household. In this study, the total household expenditure was used as the household ATP, for which higher household expenditure signified higher ATP, and vice versa. Household expenditure was a better proxy for ATP as compared to other living standard parameters, such as income, as data on income are deemed sensitive and some are reluctant to share information pertaining to their income or assets [6,17]. Another factor is that data on income tend to fluctuate among households who do not have a permanent employment or fixed income related to seasonal variations, especially among rural households engaged in small-scale industries.

Considering the household composition of adults, children and economies of scale, the household expenditure needs to be adjusted according to an adult equivalent scale (ei) [6,17] as shown in the formula:

ei = (Ai + 0.5 Ki) 0.75

where Ai is the total number of adults in a household, Ki is the total number of children in a household, and a square root of 0.75 was a recommended economy of scale used in similar study in Malaysia [16]. Subsequently, household ATP was quantified by dividing household expenditure by the adult equivalent scale (ei), which gave the estimate of monthly adult equivalent expenditure per capita, as shown in the formula:

Household ATP = Household expenditure (RM)/Adult equivalent scale (ei)

For the purpose of analysis, households’ ATP was ranked according to their respective total household expenditures. The households were ranked and classified into five quintiles of ATP, from the poorest 20% to the richest 20% (Q1 to Q5). Both variables (household OOP payments and ATP) in this study were valued according to the local currency, the Malaysian ringgit (RM). MYR 1.00 is equivalent to USD 0.24, based on the current exchange rate set by the Central Bank of Malaysia [18].

### 2.4. Progressivity Analysis of OOP Payments for Healthcare

**Figure 1:**
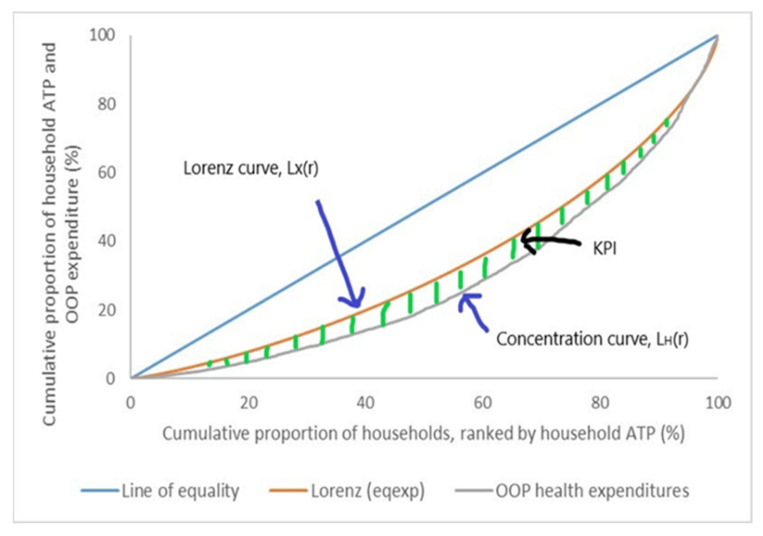
An example graph of progressive concentration curves for OOP payments. Source: author’s own work.

Progressivity is used to assess the equity of OOP payments for healthcare. Progressivity assesses the relation between household OOP payments and ATP. Progressive OOP payments are when the share or proportion of OOP payments from household ATP increase along the ATP quintiles and vice versa. In other words, it is progressive when the richer households spend more on OOP payments than the poorer households, and it is regressive when poorer households spend more on OOP payments than the richer households. The concept of the progressivity relationship between OOP payments and household ATP can be projected through plotting a concentration curve (Figure 1). On the *x*-axis, the graph shows the cumulative percentage of households ranked by increasing ATP, from poorest to richest, while the cumulative percentage of household ATP and OOP payments are on the *y*-axis. In this graph, three lines or curves can be plotted, namely:

(i) The line of equality, in which the ATP of all households are equal (45-degree blue line);
(ii) The Lorenz curve for ATP, *Lx*(*r*), plots the cumulative percentage of ATP against the cumulative percentage of the households, ranked by ATP [19,20];
(iii) The concentration curve for OOP payments, *LH*(*r*), plots the cumulative percentage of OOP payments against the cumulative percentage of the households, ranked by ATP. Two indexes can be derived from the Lorenz curve, (*Lx*(*r*), and the concentration curve for OOP payments, *LH*(*r*), which is the Gini coefficient for ATP, *Gx*, and the concentration index for OOP payments, *Ch* for the latter. The *Gx* is twice the area between the *Lx*(*r*) and the line of equality, in which *Gx* ranges from 0 to +1, where a value of +1 implies a case of perfect inequality in the distribution of ATP, while a value of 0 implies perfect equality in the ATP distribution. As for *Ch,* it is twice the area between the *LH*(*r*) and the line of equality. The *Ch* ranges from −1 to +1, where a value of −1 implies that the poorest household contributes all OOP payments and a value of +1 is where all OOP payments are made by the richest household. A negative *Ch* value is when the *LH*(*r*) lies above the line of equality, which implies that OOP payments are more concentrated among the poorer household, while a positive value is when the *LH*(*r*) lies below the line of equality, which implies that OOP payments are more concentrated among the richer household [6,7].

As for progressivity, progressive OOP payments are observed when the *LH*(*r*) lies below the *Lx*(*r*), while for a regressive OOP payment, it is the opposite: the *LH*(*r*) lies above the *Lx*(*r*). From the concentration curves, progressivity of OOP payments can be measured through the Kakwani Progressivity Index (KPI), developed by Kakwani N. [21]. From the example shown in Figure 1, the KPI is defined as twice the area between the *Lx*(*r*) and the *LH*(*r*). The formula to define KPI is as follows:

KPI = 2 ∫ [*LH*(*r*) − *Lx*(*r*)] *dr* = *Ch* − *Gx*

The range of values for KPI lies between −2 and +1. The KPI with a minimum value of −2 (in which −2 = −1 − *Gx*) indicates the most regressive distribution where, in this case, ATP is significantly higher for the richest households and all OOP payments are made by the poorest households. On the other hand, a maximum KPI value of +1 (in which +1 = 1 − *Gx*) indicates the most progressive distribution, where ATP is equally distributed from the poorest to richest households and all OOP payments are made by the richest households [6,7]. A positive KPI value (KPI > 0) implies progressive OOP payments as richer households contribute proportionately more than their share of ATP, while a negative KPI value (KPI < 0) implies regressive OOP payments, where the proportion of OOP payments made by the poorer households is greater than their share of ATP.

### 2.5. Data Analysis

The distribution of household expenditures and OOP payments for different types of healthcare services or items was analyzed descriptively in terms of its amount (in RM), proportion and mean. The distribution of OOP payments was also analyzed by its proportion of the total household expenditures, as well as across household ATP quintiles. The progressivity analysis included the plotting of the Lorenz curve for household ATP and OOP payment concentration curves, followed by calculating the Kakwani Progressivity Index (KPI). Data analysis in this study was performed using SPSS Version 23.0 and Microsoft Excel 2016 Version 16.0.

## 3. Results

### 3.1. Distribution of Household Out-of-Pocket Payments for Healthcare Services or Items in Malaysia, 2014/2015

**Figure 2:**
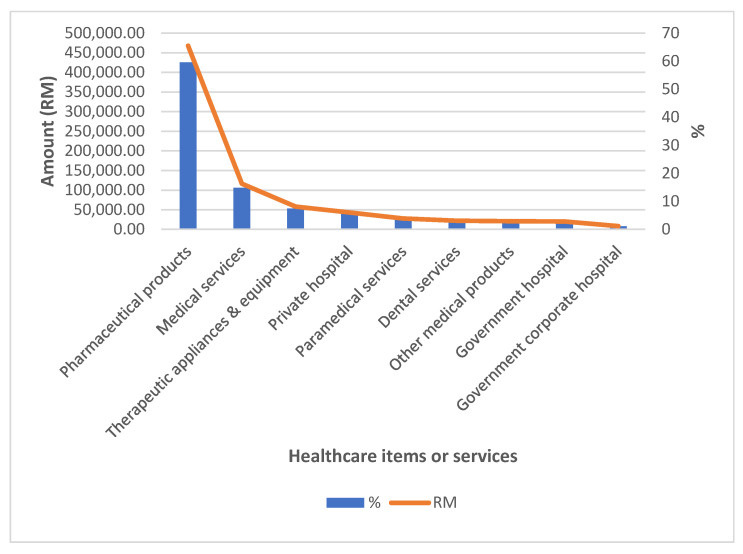
Distribution of monthly household OOP payments for healthcare services or items in HES, Malaysia 2014/2015 (*n* = 14,473).

Total monthly household expenditures in HES 2014/2015 were recorded at MYR 48,932,560.16, while the total monthly OOP payments for healthcare were recorded at MYR 784,255.94, with an average of MYR 54.18 per household. Households’ OOP payments were mostly spent on purchases of pharmaceuticals, at MYR 467,679.58 (59.6%), followed by outpatient medical services at MYR 116,386.11 (14.8%), therapeutic appliances and equipment at MYR 57,990.27 (7.4%), private hospitals at MYR 42,935.42 (5.5%), paramedical services at MYR 27,788.56 (3.5%), dental services at MYR 22,134.24 (2.8%), other medical products at MYR 20,720.04 (2.6%), government hospitals at MYR 20,095.72 (2.6%) and government corporate hospitals at MYR 8526.00 (1.1%) (Figure 2). In terms of OOP payments for groups of healthcare services or items, it was the highest for pharmaceuticals (59.6%) with a mean of MYR 32.00, while hospital-based services represented the least OOP expenditure (9.1%) with a mean of only MYR 4.68. Spending for outpatient-based services and medical products, appliances and equipment was 21.2% (with a mean of MYR 11.34) and 10.0% (with a mean of MYR 5.41), respectively.

### 3.2. Distribution and Progressivity of OOP Payments for Healthcare by Household ATP Quintiles in Malaysia, 2014/2015

**Figure 3:**
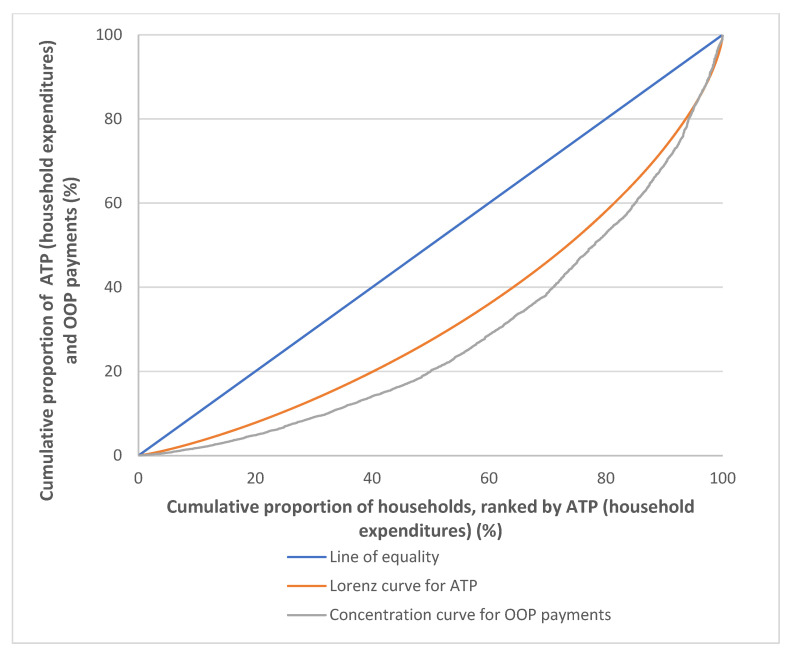
Concentration curves for OOP healthcare payments, Malaysia 2014/2015.

Overall, the proportion of OOP payments made for healthcare from the household expenditures was 1.65% (MYR 22.56 monthly per capita). The absolute amounts and proportion of OOP payments for healthcare increased from the poorest to richest household ATP quintiles. The proportion of OOP payments from household expenditures made by the richest 20% quintile (Q5) was 1.86% (MYR 53.77 monthly per capita), as compared to only 1.03% (MYR 5.55 monthly per capita) made by the poorest 20% quintile (Q1), which was almost ten times lesser (Table 1). The burden of OOP payments for healthcare among Malaysian households in 2014/2015 was more concentrated among the richer households and progressively distributed, as evidenced by the concentration curve for OOP payments, which that lies below the Lorenz curve for ATP (Figure 3).

Cumulative population shares of household expenditures and OOP healthcare payments increased along the household ATP quintiles. The richest 20% quintile (Q5) had almost half of the total household expenditure shares (42.06%), while the poorest 20% quintile (Q1) (7.72%) had less than 10% of household expenditure shares (Table 2). A similar pattern observed in shares of OOP healthcare payments, in which Q5 had the largest shares at 46.98%, as compared to Q1, which had only 4.91%. Both household expenditures and OOP healthcare payments were more concentrated among the richer households, as is evident from a positive value of the concentration index for OOP healthcare payments (0.4296). From the Gini coefficient and concentration index, it came up with a positive KPI value of 0.0910, which indicates a progressive OOP payment for healthcare in Malaysia (Table 2).

### 3.3. Distribution and Progressivity of OOP Payments among Different Group of Healthcare Services in Malaysia, 2014/2015

**Figure 4:**
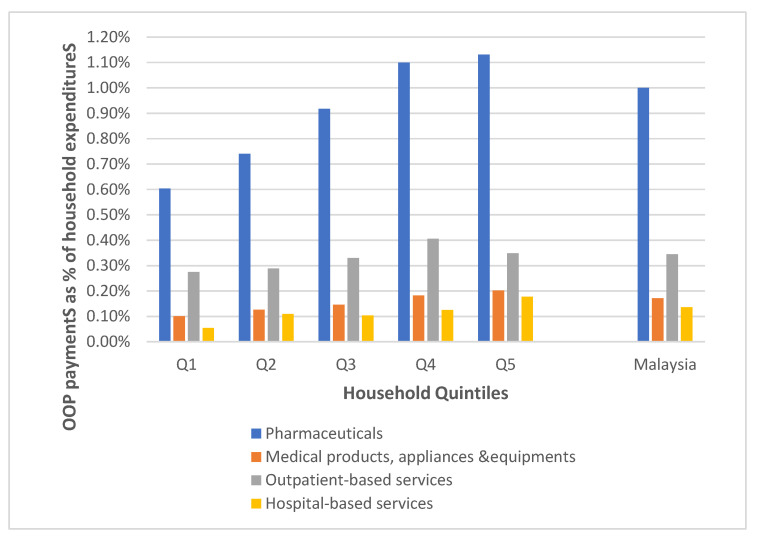
Proportion of OOP health payments from household consumption by groups of healthcare services, Malaysia 2014/2015.

The monthly per capita household OOP healthcare payments were incurred mainly from pharmaceuticals (MYR 13.65), followed by outpatient-based services (MYR 4.72), the purchase of medical products, appliances and equipment (MYR 2.34) and hospital-based services (MYR 1.86). Expenditures on hospital-based services made up the smallest portion of household OOP healthcare payments (0.15%), whereby the payments for pharmaceuticals were the highest at 13 times higher than those of hospital-based services and comprised 1% of OOP payments from the total household expenditures (Table 3). In terms of distribution of OOP payments along the household ATP quintiles, most of it was incurred from the purchase of pharmaceuticals in each of the quintiles and increased in proportions from the poorest to the richest households. A similar trend was observed for other healthcare services, such as purchases of medical products, appliances and equipment and hospital-based services, with the exception of outpatient-based services, which decreased from Q4 onward (Figure 4).

**Figure 5:**
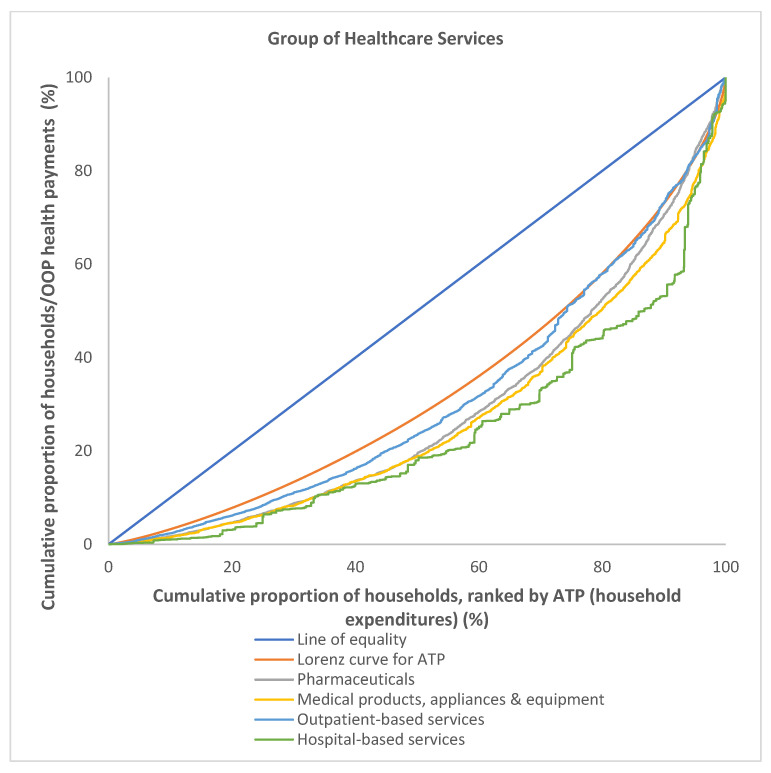
Concentration curves for OOP payments by group of healthcare services, Malaysia 2014/2015.

The OOP payments for healthcare were most concentrated among the richer households in the hospital-based services group with the highest concentration index of 0.5142, followed by medical products and appliances (0.4578) and pharmaceuticals (0.4311). In general, the OOP payments were found to be progressive for all groups of healthcare services, as the concentration curves for all groups of healthcare services lay under the Lorenz curve for ATP (Figure 5) and all groups had a positive value for KPI. However, the concentration curve for outpatient-based services was noted to cross the Lorenz curve just after the second half of Q4, leveled proportionately with the Lorenz curve for ATP. The concentration curves for OOP payments made for hospital-based services lay below the Lorenz curve for ATP and were the farthest, as compared to other group of healthcare services (Figure 5). This indicate that hospital-based services were the most progressive and concentrated among the richer households, particularly among the richest 20%, Q5. It is evident from the calculated KPI (Table 4) as hospital-based services had the highest KPI of 0.1756, followed by medical products and appliances (0.1192) and pharmaceuticals (0.0925). The least progressive group was the purchase of outpatient-based services, with a KPI of 0.0394.

## 4. Discussion

This study has shown that the OOP healthcare payments made by Malaysian households between 2014 to 2015 were progressive and more concentrated among the richer segment of the population, as is evident from the positive value of its concentration index and KPI. Progressivity studies on the mean of financing healthcare, including OOP payments, are relatively new in Malaysia, although they are quite common internationally. There were limited similar studies conducted previously based on similar HES datasets from previous years. Among the studies carried out was a study assessing Malaysia’s OOP healthcare expenditure using data from HES 1998/1999 [14] and HES 1993/1994 to HES 2004/2005 [16]. Compared to previous studies, the shares or proportion of OOP payments from the household expenditure followed an increasing pattern, as the shares were only 1.13% in 2004 as compared to 1.65% in the current study. Furthermore, the concentration index for OOP healthcare payments also declined over time, from 0.5518 (1994) to 0.5060 (1999) to 0.5034 (2005) to 0.4296, as obtained in this study (2015) [16]. All studies on OOP healthcare payments in Malaysia previously found progressive results in terms of positive and progressive KPI values [14,15,16]. However, for the last two decades, the trend of the KPI of OOP healthcare payments decreased from 0.1794 in 1994 to 0.0910 in this current study, conducted for the year 2015, suggesting decreasing progressivity. This pattern of increasing shares and decreasing progressivity of OOP healthcare payments indicates narrowing of the socioeconomic gap, in which the burden of OOP payments has shifted toward the poorer segment of the population. This finding is supported by a recent Malaysian study showing prevalent distress from OOP financing, where the poor are forced to borrow money or sell assets to pay for healthcare services [22]. However, the study by Mohd Hassan et al., (2022) utilized different datasets from the National Health and Morbidity Survey (NHMS). In contrast, our study utilized HES data, which are a more accurate measure of household ATP; therefore, household-related expenditures surveys are more commonly used in health financing studies.

Regardless of the household socioeconomic background, more and more OOP payments were made to obtain healthcare services or items, predominantly from the private healthcare service providers in the country. This was evident from the Malaysian National Health Accounts, showing that 74% of Malaysian’s OOP expenditures went to the private healthcare sectors [4]. Private OOP payment healthcare spending has also increased five-fold in two decades, from MYR 4000 million in 1997 to MYR 21,000 million in 2014. Furthermore, in a span of twenty years, there was rapid growth in the number of private healthcare facilities from 50 to 224, a period in which the number of private outpatient services almost tripled, while the hospital or inpatient services were more than doubled [23,24]. Private healthcare facilities were more preferred by Malaysians as the services are perceived to be of a better quality, highly accessible, particularly in urban areas, and have shorter waiting times as compared to similar public facilities [25,26]. Due to Malaysia’s dual-tiered healthcare system, the populations are able to choose the healthcare services of their preference [27], resulting in progressive OOP healthcare payments. Richer households who can afford it usually opted to go to costlier private healthcare facilities, whereby the poorer households, such as those from the middle- and lower-income group, utilized more public facilities, which are cheaper and heavily subsidized by the Malaysian government.

In terms of OOP payments made for different group of healthcare services, all of the OOP payments were progressive and concentrated among the richer households. Hospital-based services were most concentrated among the richer households, as evident from the highest concentration index of 0.5142, and the most progressive, with a KPI of 0.1756. Following this the purchase of medical products and appliances, with a concentration index of 0.4578 and KPI of 0.1192, while it was the least concentrated among richer households, and the least progressive value was found for outpatient-based services. This indicates that the richer households in Malaysia are paying more and prefer expensive health services, such as those provided by private hospitals, and purchase expensive medical appliances, such as vision aids, wheelchairs, blood pressure monitoring devices, etc. When compared to the previous years, household OOP expenses were highest for outpatient-based services, followed by pharmaceuticals, from 1993 to 1998. However, the trend changed in 2004 onwards, as the purchase of pharmaceuticals overtook the outpatient-based services [16]. Overall, for the past two decades from 1993 to 2014, the trend of OOP expenses saw a gradually increasing pattern for the purchase of pharmaceuticals and purchase of medical products and appliances, whereby for the expenses for hospital-based care were decreasing, and expenses for outpatient-based services decreased from 1993 to 2004 but increased again in 2014.

In terms of progressivity, the progressivity of healthcare services for all groups also decreased over the last two decades, with the expenses of hospital-based services being the most markedly reduced from 1993 (KPI of 0.3621) to 2014 (KPI of 0.1756). The KPI of OOP payments for outpatient-based services was also very much reduced from 0.1224 in 1993 to 0.0394 in 2014 within the same period. This coincides with the rapid growth of private healthcare hospitals within the same period, as well as private health clinics and laboratories [23]. The OOP payments for outpatient-based services have been the least progressive, compared to other groups of healthcare services, since 2004, as more people, regardless of their socioeconomic background, utilized private outpatient facilities, such as medical clinics, dental clinics, hemodialysis centers, etc., due to their convenience, higher-quality services and accessibility [25,26,28]. There was also a mismatch in terms of distribution of healthcare resources, quantity of health facilities and workloads between the public and private healthcare facilities in Malaysia, resulting the healthcare resources being sub-optimally utilized. As of 2010, there were 209 licensed private hospitals and only 130 government hospitals, while the number of private medical clinics was at 6371, as compared to only 808 public health clinics. There was also a workload discrepancy between public and private healthcare services; in such a case, the public primary healthcare sector had only 10% primary care clinics, but handled almost half (45%) of Malaysian outpatient visits [24].

The progressivity of Malaysia’s OOP healthcare payments is internationally comparable. If compared with regional neighbors, Malaysia’s OOP healthcare payments were more progressive than Indonesia’s OOP payments (KPI between 0.05 and 0.04) [29]. Similar to Malaysia, the trend of progressivity of OOP payments in Indonesia also decreased over the years. However, OOP payments were not the only source of healthcare financing in the country, as Indonesia incorporated mandatory social health insurance schemes known as Jaminan Kesihatan Nasional (JKN) in 2014 to reduce the OOP spending among its households. Another neighboring country, Thailand, also has consistent progressive OOP payments throughout the years with a KPI ranging from 0.26 to 0.28 [30]. Furthermore, in 2001, Thailand introduced mandatory social health insurance known as the Universal Coverage Scheme (UCS) in their healthcare financing mix, which significantly reduces the country’s incidence of catastrophic health expenditures (CHE) and impoverishment due to OOP payments [31,32].

Progressivity of OOP healthcare payments varies in other countries within the Asian continent. The OOP payments were progressive in other upper-middle income countries, such as China, similar to Malaysia, the OOP payments were mostly concentrated among the richer households and increased along the household ATP [11]. However, in Iran, the OOP payments were regressive, although the regressivity trend decreased over time, but increasing incidence of CHE indicates inequitable OOP payments [33,34]. In lower-middle income Asian countries, such as India and Bangladesh, the OOP healthcare payments there were generally regressive [9,10]. One of the reasons for regressive OOP payments in those countries was that a high proportion of OOP payments were made by the poorer segment of the population, especially in rural areas where access to healthcare and health protection schemes for the poor are lacking. The regressivity of OOP payments in India was also attributable to high OOP spending on private healthcare services among its population, particularly for inpatient services, which are perceived as superior to the public care [35].

The OOP healthcare payments tend to be regressive in more developed Asian countries. Taking the Republic of Korea as an example, their overall healthcare financing system was regressive, not only for OOP payments, but also for its National Health Insurance and private health insurance scheme. This was due to high OOP payments made by their population regardless of their socioeconomic status as copayments for healthcare services that are not covered under either national and private health insurance, such as for expensive oncological medications and diagnostics [36]. The same scenario can be seen in other developed European countries, such as Austria, in which the OOP healthcare payments were regressive. High OOP payments were made as copayments for expensive drugs and healthcare services that were not covered by the health insurance. The most regressive OOP payments were made for pharmaceuticals, including both prescriptions and over-the-counter (OTC) medicines [37]. The OOP payments for healthcare in Italy were also regressive, and the regressivity was much more pronounced in the socioeconomically poorer southern region [38].

## 5. Study Limitations

This study used secondary data obtained from the HES 2014/2015 provided by the Department of Statistics. Since this a household population survey, the HESs are prone to non-sampling errors such as bias, which could affect the data quality and accuracy. Possible biases were recall bias in which respondents were unable to recall the exact amount of household expenditures made for OOP payments and other non-health household items. Respondents may also withhold or fabricate sensitive information about income, assets or household expenditures as they were not required to produce income tax records or purchase receipts during the survey. With regard to the average household’s OOP health payments, the value obtained does not represent the whole population in this study as a high amount in OOP payments might have come from a few households with catastrophic health expenditure (CHE) or those that were impoverished because of the health spending. CHE and impoverishment from OOP payments are other equity indicators which were not studied in this paper. The analysis of households’ OOP payments in this study did not include payments made to private health insurance, as many studies define private health insurance as another source of healthcare financing on top of OOP payments [29,38]. Furthermore, in HES 2014/0215, only a small portion of households (8%) reported to have expenses for private health insurance.

## 6. Conclusions

In conclusion, the OOP payments for healthcare made by Malaysian households in 2014/2015 were progressively distributed and equitable as they were more concentrated among the richer households. This is partly due to Malaysia’s dual-tiered healthcare system, where, regardless of the socioeconomic status, the population has a choice in accessing healthcare services between costlier private healthcare facilities or more affordable public healthcare facilities. However, the progressivity trends of OOP health payments have decreased steadily over time as compared to previous national studies. This alarming trend should be closely monitored by the policymakers. Findings from this study can provide insight for policymakers into the current situation of Malaysia’s OOP health payments, and based on it, strategies on policy improvements to cater to the health needs of Malaysian households can be developed accordingly, especially for the poorer households, who are more vulnerable to financial risk catastrophe. There is also a need to conduct a similar study using the latest Malaysian HES data to analyze the progressivity trend of OOP health payments in the country. In view of how high OOP payments can negatively affect financial risk protection, further studies on catastrophic health expenditure and the impoverishing effect of OOP payments are recommended to complement the findings from this study.

## Figures and Tables

**Table 1:** Household expenditures and OOP payments for healthcare by household ATP quintiles, Malaysia 2014/2015.

Household ATP Quintiles	Per Capita Household Expenditure ^1^ (MYR *)	Per Capita OOP Payments ^2^ (MYR *)	OOP Payments as % of Household Expenditures
Q1	536.86	5.55	1.03
Q2	830.60	10.51	1.27
Q3	1111.26	16.65	1.50
Q4	1517.49	27.51	1.81
Q5	2891.77	53.77	1.86
Total population	1365.93	22.56	1.65

^1^ Refers to monthly per capita household expenditure. ^2^ Refers to monthly per capita household OOP payments for healthcare. * MYR 1.00 is equivalent to USD 0.24.

**Table 2:** Cumulative population shares, Gini coefficient, concentration index and Kakwani Progressivity Index of OOP payments for healthcare, Malaysia 2014/2015.

Household ATP Quintiles	Household Expenditures (%)	OOP Healthcare Payments (%)
Q1	7.72	4.91
Q2	12.27	9.13
Q3	16.13	14.87
Q4	21.82	24.11
Q5	42.06	46.98
Total population	100.00	100.00
Gini coefficient/Concentration index	0.3386	0.4296
Kakwani Progressivity Index		0.0910

**Table 3:** Household expenditures and OOP payments by groups of healthcare services, Malaysia 2014/2015.

Groups of Healthcare Services	Per Capita OOP Payments ^1^ (MYR *)	OOP Payments as % of Household Expenditures
Pharmaceuticals	13.65	1.00
Medical products, appliances and equipment	2.34	0.17
Outpatient-based services	4.72	0.35
Hospital-based services	1.86	0.15
Total population	22.56	1.65

^1^ Refers to monthly per capita household OOP payments for healthcare. * MYR 1.00 is equivalent to USD 0.24.

**Table 4:** Gini coefficient, concentration index and Kakwani Progressivity Index for OOP payments for healthcare by groups of healthcare services, Malaysia 2014/2015.

Groups of Healthcare Services	Household Consumption	OOP Health Payments
Pharmaceuticals		
Gini coefficient/Concentration Index	0.3386	0.4311
Kakwani Progressivity Index		0.0925
Medical products, appliances and equipment		
Gini coefficient/Concentration Index	0.3386	0.4578
Kakwani Progressivity Index		0.1192
Outpatient-based services		
Gini coefficient/Concentration Index	0.3386	0.3780
Kakwani Progressivity Index		0.0394
Hospital-based services		
Gini coefficient/Concentration Index	0.3386	0.5142
Kakwani Progressivity Index		0.1756

## Data Availability

Not applicable.
